# State-of-the-Art Perspectives on Postbiotic-Oriented Systems Derived from Fermented Medicinal Plant Extracts

**DOI:** 10.3390/foods15050864

**Published:** 2026-03-04

**Authors:** Vanja Travičić, Lato Pezo, Mirjana Sulejmanović, Dina Tenji, Milica Perović, Gordana Ćetković, Nenad Ćetković

**Affiliations:** 1 Faculty of Technology Novi Sad, University of Novi Sad, Bulevar Cara Lazara 1, 21000 Novi Sad, Serbia; mirjana.sulejmanovic@uns.ac.rs (M.S.); perovicmilica@uns.ac.rs (M.P.); gcetkovic@uns.ac.rs (G.Ć.); 2 Institute of General and Physical Chemistry, University of Belgrade, 11000 Belgrade, Serbia; latopezo@yahoo.co.uk; 3 Faculty of Sciences, University of Novi Sad, Trg Dositeja Obradovića 3, 21000 Novi Sad, Serbia; dina.tenji@dbe.uns.ac.rs; 4 Medical Faculty, University of Novi Sad, Hajduk Veljkova 3, 21000 Novi Sad, Serbia; nenad.cetkovic@mf.uns.ac.rs

**Keywords:** medicinal plants, fermentation, LAB, biotransformation, postbiotics, phytochemicals

## Abstract

Fermentation is increasingly used as a controlled bioprocessing approach to modify medicinal plant extracts by selectively transforming phytochemicals while maintaining safety and compositional integrity. Controlled in vitro fermentation has gained attention as a practical method to generate stable, cell-independent bioactivity consistent with postbiotic concepts. This review examines Scopus-indexed studies on fermented medicinal plant extracts, focusing on microbial platforms, fermentation strategies, dominant biotransformation pathways, and functional outcomes. Evidence indicates that fermentation is not a uniform process but follows platform-specific enzymatic pathways that reshape phenolics, flavonoids, alkaloids, and polysaccharides. Lactic acid bacteria (LAB) are most frequently applied due to their safety profile and enzymatic capacity, while yeasts and filamentous fungi enable complementary matrix restructuring and deeper chemical modification. Across systems, fermentation-driven biotransformation produces bioactive profiles that persist independently of microbial viability, supporting a postbiotic-oriented interpretation. Applications have been reported in food, nutraceutical, cosmetic, and animal nutrition contexts, although clinical translation remains limited. Remaining challenges include incomplete mechanistic understanding, limited standardization, and unclear regulatory positioning.

## 1. Introduction

Medicinal plants have long been recognized as essential sources of bioactive compounds, forming the basis of traditional medical systems and contributing to modern pharmacotherapy. Specifically, the World Health Organization (WHO) estimates that approximately 80% of the global population relies partially on plant-based medicinal preparations for primary healthcare needs [1]. Their therapeutic relevance is rooted in a chemically complex phytochemical composition, including polyphenols, terpenoids, alkaloids, glycosides, and structurally diverse polysaccharides. However, substantial evidence indicates that the biological efficacy of medicinal plants is often limited by the molecular form and bioaccessibility of their compounds [2,3].

In native or conventionally extracted medicinal plant materials, a significant amount of bioactive compounds occurs in glycosylated or polymerized forms, which exhibit limited intestinal absorption and reduced biological activity. For example, flavonoids such as quercetin, luteolin, and apigenin are predominantly present as glycosides, while iridoids and saponins often require enzymatic cleavage to exert pharmacological effects [4,5]. These limitations have caused increasing interest in processing strategies capable of selectively modifying phytochemical structure while preserving safety and compositional integrity. Comparable enzyme-driven structural modifications have also been reported in non-plant biological matrices, further illustrating how controlled enzymatic activity can reshape the molecular architecture and functional properties of complex systems [6].

Microbial fermentation has emerged as one of the most promising approaches for these. While traditionally associated with the preservation and sensory modification of food, fermentation is now increasingly recognized as a controlled bioprocessing strategy capable of reshaping plant phytochemical profiles through targeted microbial metabolism [7,8,9]. This advance is particularly relevant for medicinal plant extracts, which provide concentrated, chemically defined substrates that allow precise control over fermentation parameters, microbial strains, and processing conditions [5]. Unlike whole fermented foods, extract-based systems enable reproducible investigation of microbial–phytochemical interactions and their conceptual consequences.

A growing number of studies highlight that the fermentation of medicinal plant extracts facilitates systematic biotransformations, resulting in verified functional changes. For instance, fermentation of *Glycyrrhiza uralensis* extracts with lactic acid bacteria (LAB) has been shown to convert glycosylated flavonoids into aglycone forms with significantly enhanced antioxidant and anti-inflammatory activity [7]. Similarly, controlled LAB-mediated fermentation of *Panax ginseng* extracts has been shown to induce stepwise deglycosylation of ginsenosides, yielding low-molecular-weight metabolites with enhanced bioavailability and immunomodulatory activity [10]. Comparable fermentation-driven transformations have been reported for other classes as well. LAB-mediated biotransformation of flavonoids from *Scutellaria baicalensis* has been demonstrated under controlled fermentation conditions, resulting in structurally simplified aglycones with enhanced functional activity [11]. Additionally, controlled fermentation of polysaccharide-rich extracts from *Astragalus membranaceus* has been shown to modify polysaccharide structure and molecular-weight distribution, generating fermentation-derived fractions with enhanced immunostimulatory activity [12].

Meanwhile, the concept of postbiotics has gained prominence as a unifying framework for interpreting fermentation-derived bioactivity. The ISAPP consensus defines postbiotics as preparations of inanimate microorganisms and/or their components that confer a health benefit on the host [13]. It is important to note that this framework extends beyond microbial cell fragments to include fermentation-derived metabolites, cell-free supernatants, exopolysaccharides, peptides, and structurally transformed phytochemicals, provided that biological activity is demonstrated independently of microbial viability [14].

Fermented medicinal plant extracts represent a significant platform for postbiotic generation. LAB dominate this field due to their qualified presumption of safety (QPS) status, metabolic versatility, and ability to express enzymes such as β-glucosidases, esterases, phenolic acid decarboxylases, and proteases [7,15]. These enzymatic systems enable phytochemical biotransformation, as well as the accumulation of microbial-derived postbiotic fractions, including organic acids, exopolysaccharides, and bioactive peptides. Such components have been shown to modulate oxidative stress, inflammatory signalling, epithelial barrier integrity, and microbial homeostasis in in vitro and ex vivo models, even in the absence of viable microorganisms [14,15].

This review examines Scopus-indexed studies on the fermentation of medicinal plant extracts, focusing on how different microorganisms and fermentation approaches influence chemical transformations and biological effects. Through a structured comparative evaluation of microbial platforms, dominant biotransformation pathways, and the level of chemical and biological validation, the review demonstrates that fermentation does not act as a single, uniform process. Instead, it represents a platform-specific bioprocess that reshapes phytochemical structure and generates stable bioactive profiles capable of persisting independently of living cells. This supports the view within the reviewed literature of fermented medicinal plant extracts as postbiotic-oriented functional systems.

## 2. Literature Search Strategy and Study Selection

**Figure 1:**
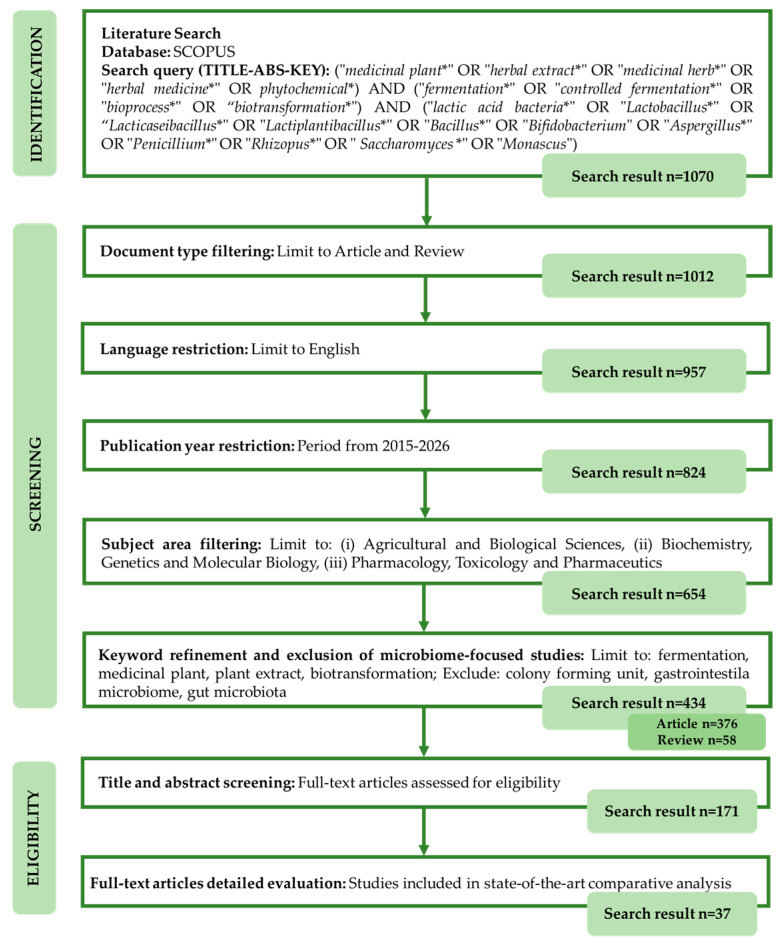
Flow diagram illustrating the literature search and study selection process based on the Scopus database.

In January 2026, the Scopus database was systematically searched to identify peer-reviewed publications related to the controlled fermentation and biotransformation of medicinal plants and herbal extracts. A structured search strategy was applied, and the resulting records were sequentially filtered based on document type, language, publication period, subject area, and topical relevance. The selection process involved an initial title and abstract screening, followed by a full-text evaluation to identify studies suitable for state-of-the-art comparative analysis. The complete workflow of literature identification, screening, eligibility assessment, and final study inclusion is summarized in Figure 1.

To ensure transparent and reproducible study selection, predefined inclusion and exclusion criteria were applied at both the title and abstract screening stage and the full-text evaluation stage. These criteria were designed to refine the dataset by retaining studies directly relevant to controlled fermentation or biotransformation of medicinal plants, while excluding publications lacking clear microbial involvement, fermentation-driven transformation, or sufficient methodological detail. The specific criteria applied at each selection stage are summarized in Table 1.

### Bibliometric Co-Occurrence Analysis

A bibliometric co-occurrence analysis was conducted using abstracts from the articles retained after title and abstract screening and before full-text assessment (*n* = 171). Abstracts were compiled into a single corpus, and terms were standardized by merging synonymous expressions and removing irrelevant or redundant phrases to improve analytical consistency. Only phrases related to microbial sources, fermentation processes, bioactive compounds, analytical approaches, and functional or health-related outcomes were retained.

The co-occurrence network of main phrases was generated using VOSviewer software (version 1.6.20) (Leiden University, The Netherlands). A full counting method and association strength normalization were applied. A minimum occurrence threshold was set, and a minimum cluster size of 10 terms was used for clustering. Network visualization was performed at a resolution of 1000 dpi. In the resulting map, nodes represent phrases, with node size proportional to occurrence frequency, while links indicate co-occurrence relationships, with link thickness reflecting total link strength. Clustering was performed using the VOSviewer algorithm, with each cluster displayed in a distinct colour. The spatial proximity and interconnections among clusters were interpreted as indicators of conceptual overlap among fermentation processes, bioactive compound characterization, and functional and health-oriented applications, highlighting the multidisciplinary nature of the reviewed literature.

Recent advances in food biotechnology and natural product research have increasingly emphasized microbial fermentation and plant-associated microorganisms as sustainable sources of bioactive compounds. To systematically characterize the intellectual structure of this research field, a bibliometric content analysis was conducted using the main phrases extracted from the abstracts of the studies included in this review. Based on phrase co-occurrence relationships and total link strength, the identified terms were grouped into seven thematic clusters, each representing a distinct but interconnected research focus.

**Figure 2:**
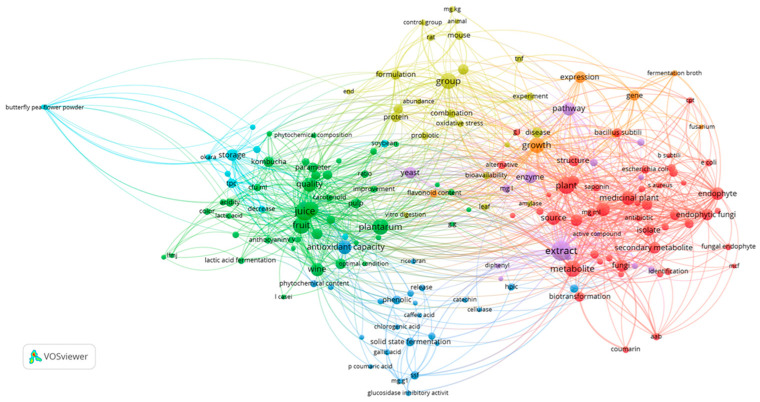
Co-occurrence network of main phrases extracted from abstracts, visualized using VOSviewer software.

A co-occurrence network visualization of the main phrases extracted from the abstracts of the reviewed studies was generated using VOSviewer software (Figure 2). In the network map, nodes represent individual phrases, while links indicate their co-occurrence relationships across the dataset, with link thickness reflecting total link strength. The size of each node is proportional to the number of occurrences of the corresponding phrase. Based on the VOSviewer clustering algorithm, the phrases were grouped into seven distinct clusters, each depicted by a different colour, representing coherent thematic areas within the literature.

Prominent clusters are centred on endophytic microorganisms and antimicrobial secondary metabolites, fermented plant-based beverages and quality attributes, fermentation-driven enhancement of phenolic compounds and antioxidant activity, in vivo functional evaluations and health-related outcomes, enzymatic biosynthesis and bioactive extract production, storage and nutritional stability of fermented products, and microbial growth and molecular regulation. The spatial proximity and interconnections among clusters indicate substantial overlap between fermentation processes, bioactive compound characterization, and functional and health-oriented applications, underscoring the multidisciplinary nature of the reviewed research.

Cluster 1 (red-highlighted group in Figure 2) is dominated by terms related to endophytic fungi and bacteria, their isolation from medicinal plants, and their antimicrobial potential. High-occurrence terms such as plants, medicinal plants, endophytes, endophytic fungi, fungi, and source indicate that the reviewed studies primarily focus on plants as reservoirs of bioactive endophytes. The strong presence of secondary metabolites, biotransformation, structure, and yield reflects an emphasis on metabolite production and characterization. Antimicrobial relevance is underscored by frequent mentions of antimicrobial activity, antibacterial activity, inhibition, and specific pathogens (*Escherichia coli*, *Staphylococcus aureus*, *Pseudomonas aeruginosa*, and *Bacillus subtilis*).

Cluster 2 (the green-highlighted group in Figure 2) captures research on functional beverages, particularly those derived from fruits and plant substrates. Highly connected terms such as juice, fruit, beverage, wine, and kombucha highlight fermentation-based beverage systems as a major topic. The frequent appearance of *Lactobacillus plantarum*, lactic acid fermentation, and lactic acid reflects the central role of lactic acid bacteria in these processes. Quality and consumer-oriented attributes are strongly represented through terms like sensory attribute, flavour, colour, quality, and shelf life, while chemical and nutritional aspects are captured by the total phenolic content, total flavonoid content, anthocyanin, carotenoid, and volatile compound.

Cluster 3 (the blue-highlighted group in Figure 2) centres on phenolic compounds and antioxidant properties, primarily in fermented matrices. Dominant terms such as antioxidant capacity, phenolic, phenolic content, and phytochemical content indicate that antioxidant evaluation is a core research objective. The frequent identification of individual compounds (gallic acid, caffeic acid, chlorogenic acid, quercetin, and catechin) reflects detailed chemical profiling, often supported by HPLC analysis.

Process-related terms (fermentation time, day fermentation, solid-state fermentation, SSF, and starter culture) highlight the role of fermentation conditions in modulating bioactive compound release and transformation. The presence of glucosidase inhibitory activity further suggests interest in functional bioactivities beyond antioxidant effects, particularly those relevant to metabolic health.

Cluster 4 (the yellow-highlighted group in Figure 2) represents studies that move beyond compositional analysis toward biological validation and functional formulation. The frequent use of terms such as animal, rat, mouse, control group, and experiment indicates in vivo experimental designs. Health-related endpoints are emphasized through terms such as disease, oxidative stress, TNF, and diet, pointing to investigations of physiological and inflammatory responses. Terms like formulation, stability, combination, and protein suggest efforts to develop stable functional products, while probiotic and bioavailability reflect interest in the efficacy and delivery of bioactive components. This cluster reflects a translational research direction linking fermented or bioactive products to measurable health outcomes.

Cluster 5 (the purple-highlighted group in Figure 2) focuses on biochemical and enzymatic mechanisms underlying bioactive compound production. Highly connected terms such as extract, enzyme, pathway, biosynthesis, and synthesis indicate mechanistic studies aimed at understanding how bioactive compounds are formed. Antioxidant and cytotoxic screening methods are reflected by terms such as picrylhydrazyl, ABTS, and cytotoxicity. The prominence of yeast and *Aspergillus niger* suggests that microbial systems are frequently employed as biocatalysts.

Cluster 6 (the cyan-highlighted group in Figure 2) groups terms related to post-fermentation handling and stability. The strong occurrence of storage indicates that shelf life and preservation effects are central concerns. Nutritional quality is reflected by nutritional value, TPC, TFC, and ABTS, while terms such as significant difference and decrease suggest comparative analyses over time or between treatments. Substrates such as soybean, okra, and products like kefir indicate that this cluster addresses diverse fermented food matrices, with attention to how storage conditions influence antioxidant retention and nutritional attributes.

Cluster 7 (the orange-highlighted group in Figure 2) captures a more molecular and microbiological perspective. Dominant terms such as growth, gene, expression, and fermentation broth point to studies investigating microbial dynamics and regulation during fermentation. The inclusion of root and *Fusarium* suggests that some studies extend into plant–microbe interactions, particularly at the rhizosphere or endophytic level. The frequent occurrence of flavonoid content within this cluster indicates that molecular-level changes are often linked to downstream effects on metabolite accumulation, bridging mechanistic biology with functional outcomes.

## 3. Conceptual Framework Linking Fermentation, Biotransformation, and Postbiotic Outcomes in Medicinal Plant Extracts

In current research on medicinal plants, fermentation, biotransformation, and postbiotics are frequently used as overlapping terms, even though they describe distinct analytical layers of the same system. Fermentation refers primarily to a controlled technological process in which selected microorganisms are applied to plant materials or extracts under defined conditions to modify their chemical composition and functional potential. In medicinal plant systems, fermentation is typically performed in vitro, before application, and is not intended to promote microbial colonization or viability in the host. This concept differs fundamentally from gastrointestinal fermentation and from the probiotic-centred model, where live microorganisms constitute the functional entity [7,8].

The principal scientific value of fermentation in medicinal plant research lies in the microbial capacity to modify phytochemical profiles through biotransformation processes. Biotransformation is generally understood as a set of enzymatically mediated reactions, including deglycosylation, hydrolysis, de-esterification, oxidation–reduction, and partial depolymerization, that may occur during controlled fermentation. Beyond phytochemical remodelling, fermentation has also been explored as a detoxification strategy, including the reduction in aflatoxin B1 in cereal-based matrices, further illustrating the broader molecular transformation capacity of microbial bioprocessing under controlled conditions [16]. Evidence from plant bioprocessing studies consistently indicates that fermentation predominantly induces qualitative compositional shifts rather than increasing total compound abundance, often resulting in lower-molecular-weight or structurally simplified derivatives with altered physicochemical and biological properties [5,7,17]. This conceptual distinction is important, as changes in bioactivity are frequently observed even when total phenolic or flavonoid content remains unchanged or decreases, suggesting that molecular form and structural context may play a significant role in determining functional outcomes.

While fermentation defines the technological process and biotransformation describes the underlying molecular mechanisms, neither concept alone captures the functional relevance of the resulting preparations. Hence, a postbiotic-oriented perspective has been proposed as a useful framework to evaluate fermentation outcomes instead of microbial viability. In fermented medicinal plant systems, this entails prioritizing cell-free extracts, metabolite-enriched fractions, and heat-treated preparations, in which bioactivity persists independently of live microorganisms. Such characteristics are frequently discussed in relation to postbiotic concepts, particularly in earlier literature that predates standardized postbiotic terminology [12,18]. Recent systematic analyses have further highlighted the capacity of probiotic-derived postbiotics to reduce aflatoxin B1 in vitro, underscoring the broader functional relevance of cell-independent microbial metabolites in detoxification contexts [19]. Within this framework, fermented medicinal plant extracts can be understood as postbiotic-oriented preparations whose functional effects arise from the combined contribution of biotransformed phytochemicals, fermentation-derived microbial metabolites, and, where present, non-viable microbial components, without invoking assumptions related to microbial survival or gastrointestinal colonization.

## 4. State-of-the-Art Analysis of Microbial Platforms, Biotransformation Pathways, and Postbiotic-Oriented Functional Outcomes in Fermented Medicinal Plant Substrates

Across the Scopus-selected literature, it becomes evident that microbial identity represents a decisive determinant of fermentation outcomes in medicinal plant systems. Fermentation does not function as a uniform or nonspecific processing step; distinct microbial platforms impose characteristic metabolic and enzymatic logics that shape biotransformation pathways, reshape phytochemical profiles, and ultimately define postbiotic-oriented bioactivity. Table 2 provides an overview of these relationships considering medicinal plant substrates, microbial platforms, dominant biotransformations, and functional outcomes reported in the literature. However, the strength of experimental evidence varies substantially across studies. While many reports rely primarily on in vitro chemical antioxidant assays and compositional profiling, a smaller number of studies incorporate cellular, mechanistic, or in vivo validation. Accordingly, functional outcomes discussed below are interpreted in relation to their level of biological support.

LAB represents the most extensively investigated microbial platform in fermented medicinal plant research and consistently emerges as a precision biotransformation agent across chemically diverse plant matrices. In LAB-driven fermentations, the fermentation process is typically characterized by controlled acidification, enzymatic activity targeting conjugated plant metabolites, and selective remodelling of existing phytochemicals.

Phenolic- and flavonoid-rich medicinal plants constitute the most frequent substrates for LAB fermentation. Across multiple studies summarized in Table 2, LAB fermentation leads to reproducible shifts in phenolic and flavonoid profiles, which are frequently associated with enhanced antioxidant capacity in chemical assays and, in selected cases, with validated cellular or in vivo effects. These changes are commonly interpreted in relation to the potential activity of LAB-expressed β-glucosidases and esterases, which are known to cleave glycosidic and ester bonds under appropriate conditions. However, in most cases, enzyme activity is inferred from metabolite redistribution, and the link between specific structural modifications and improved bioaccessibility or biological activity remains largely correlative.

For example, fermentation of *Centella asiatica* extract with *Lactobacillus plantarum* results in modified phenolic and flavonoid compositions accompanied by enhanced anti-inflammatory activity [20], while LAB-fermented *Malva sylvestris* extract exhibits increased antioxidant capacity as determined by in vitro chemical assays [20]. Despite differences in their native phytochemical composition, these systems converge functionally, indicating that LAB-driven fermentation operates through shared transformation principles across structurally distinct phenolic matrices.

Similar trends are observed in mixed or structurally complex phytochemical systems. LAB fermentation of milk thistle and liquorice root extracts induces flavonolignan and triterpenoid remodelling, leading to enhanced antioxidant and functional effects [27]. In *Orthosiphon stamineus* leaves, LAB-assisted fermentation increases the functional availability of polyphenols, an effect attributed to fermentation-mediated release and profile redistribution [38]. Generally, these examples demonstrate that LAB fermentation consistently translates phytochemical remodelling into measurable biological effects.

LAB-mediated fermentation is not limited to phenolic systems but extends to alkaloid-containing and metabolically active medicinal plants. In *Berberis vulgaris* root, LAB fermentation reshapes alkaloid–phenolic profiles associated with berberine-related pathways, resulting in increased antioxidant capacity in chemical assays and reported antimicrobial activity [21]. Although full enzymatic resolution is rarely reported, the observed changes are consistent with microbial deconjugation reactions and oxidoreductase-mediated modulation of isoquinoline alkaloid-associated chemical equilibria. In saponin-rich matrices, such as *Astragalus membranaceus* radix, LAB fermentation selectively remodels polysaccharide and saponin fractions, yielding extracts with enhanced antioxidant capacity and reported immunomodulatory potential, primarily demonstrated in in vitro assays [25]. These effects are attributed to LAB-derived glycosidases and polysaccharide-modifying enzymes, which induce partial depolymerization, molecular-weight redistribution, and increased solubility of macromolecular fractions.

Likewise, fermentation of *Amomum xanthioides* with *Lactobacillus casei* produces metabolically active preparations capable of ameliorating metabolic dysfunction in high-fat-diet-induced obese mice [26]. These in vivo findings provide stronger translational support compared to in vitro antioxidant measurements; however, confirmation of microbial inactivation and full independence from microbial viability is not consistently demonstrated across studies.

LAB-driven effects on macromolecular fractions further reinforce the versatility of this platform. Fermentation of okra juice with LAB induces polysaccharide depolymerization and structural modification, leading to improved immunomodulatory activity in cell-based assays [34]. In *Schisandra sphenanthera* fruit fermented with *Lactiplantibacillus plantarum*, polysaccharide release and restructuring underpin enhanced functional polysaccharide properties. A comparable pattern is observed in *Phyllostachys glauca* leaf juice fermented with *Streptococcus thermophilus*, where polysaccharide restructuring occurs alongside broader phytochemical changes, illustrating simultaneous remodelling of multiple matrix components [43].

Multi-strain LAB fermentations further expand functional outcomes. The presence of multiple LAB strains broadens the enzymatic potential available during fermentation, enabling the simultaneous transformation of phenolics, polysaccharides, and nitrogenous compounds. A rose–shiitake blended beverage fermented with multiple LAB strains exhibits increases in phenols, flavonoids, free amino acids, and antioxidant capacity [40], while LAB co-fermentation of wolfberry–longan juice similarly improves phenolic/flavonoid content and redox-linked bioactivity, primarily demonstrated in chemical assays [46]. These systems highlight how microbial diversity can expand functional gains without abandoning controlled fermentation logic.

Several LAB-related studies further strengthen mechanistic interpretation by linking fermentation-induced biotransformation to defined bioassay outcomes. Fermentation of *Lonicera japonica* leaves with *Lacticaseibacillus rhamnosus* increases polyphenols and flavonoids while enhancing xanthine oxidase inhibition [28]. These findings support the hypothesis of a mechanistic link between enzymatic deglycosylation of phenolics and pathway-relevant functional inhibition. Anti-pigmentation effects achieved by fermenting seven traditional *Chinese herbal* extracts with *Lactobacillus rhamnosus* and modulation of the CREB/MITF/tyrosinase pathway provide additional evidence that LAB fermentation can yield highly specific signalling-level outcomes beyond generic antioxidant claims [54]. Still, to define the general antioxidant effect, further specific pathway validation is required.

Yeast-based fermentation platforms, predominantly involving *Saccharomyces* species, display a distinct fermentation logic compared to LAB systems. Yeast systems rely on esterases, alcohol dehydrogenases, and redox-active enzymes that modulate secondary metabolite distribution, organic acid profiles, and volatile compounds, rather than inducing them.

Fermentation of *Leonurus japonicus* with *Saccharomyces cerevisiae* results in phenolic remodelling associated with protection against UVB-induced skin damage, positioning yeast fermentation as a promising strategy for cosmetic-relevant medicinal plant applications [22]. Similarly, fermentation extraction of *Orthosiphon stamineus* leaves with *Saccharomyces cerevisiae* increases polyphenol-linked antioxidant capacity determined by in vitro redox assays, consistent with yeast-mediated release and transformation mechanisms [38]. In saffron petals (*Crocus sativus*), *Saccharomyces cerevisiae* fermentation increases anthocyanin levels while reducing amino acid content, illustrating that yeast platforms can shift multiple compositional axes simultaneously, with implications for both bioactivity and formulation design [31].

Mixed-yeast systems further expand the potential for metabolic biotransformation. For instance, the fermentation of *Elaeocarpus sylvestris* hot-water extracts with *Pichia fermentans* and *Saccharomyces cerevisiae* significantly alters organic acid and oxygenated compound profiles [36]. This emphasizes that yeast platforms can effectively reshape non-phenolic metabolite fractions, which contribute significantly to the functional properties and sensory attributes of the final extract.

Filamentous fungi represent a powerful biotransformation, enabling deep structural modification and diversification of phytochemicals. This capacity arises from their extensive enzymatic compounds, including monooxygenases, hydroxylases, dehydrogenases, and diverse glycosidases capable of regioselective and stereoselective reactions.

For example, *Dendrobium officinale* leaves fermented with *Aspergillus oryzae* show increased levels of phenolic aglycones, such as quercetin, and enhanced bioactivity, reflecting the fungus’s ability to process glycosides [33]. Similarly, *Aspergillus niger* fermentation of moringa leaf flour increases phenolic and flavonoid content while generating new antioxidant and antimicrobial compounds, proving that fungal fermentation is an effective route for diversifying chemical compositions [52]. Solid-state fermentation of *Diaphragma juglandis* fructus with *Aspergillus niger* also boosts polyphenol levels and biological activity, highlighting how solid-state fermentation improves enzymatic efficiency by promoting better contact between the fungi and the plant matrix [35].

Fungal platforms are also capable of specifically targeting and changing the key active compounds in plants. For example, fermenting wild turmeric (*Curcuma aromatica*) with *Rhizopus oligosporus* selectively increases curcuminoid levels and antioxidant capacity as assessed in chemical assays [50]. Similarly, when *Andrographis paniculata* is processed by *Aspergillus oryzae*, it produces new types of diterpenoids through chemical reactions like oxidation and hydroxylation [55]. These cases show that fermentation is a controlled way to modify or create entirely new medicinal compounds, rather than just releasing what is already in the plant.

Bacterial solid-state fermentation also helps make medicinal plants safer to use. For instance, when *Ginkgo biloba* leaves are fermented with *Bacillus subtilis* and treated with pulsed light, the levels of toxic ginkgolic acids are significantly reduced [29]. At the same time, the beneficial compounds are preserved or even increased. This shows that fermentation can act as a detoxification step while keeping the plant’s health benefits intact. This approach is especially useful for postbiotic products, which offer better safety and stability than products containing live bacteria.

Specifically, using multiple types of microbes together shows how different fermentation strategies can lead to the same goal. Fermenting ginger with *Bifidobacterium adolescentis* and *Monascus purpureus* increases its flavonoid levels and antioxidant power as measured in chemical assays [30]. Similarly, mulberry-based systems using various fungi (*Monascus* species) consistently lead to better flavonoid diversity and stronger anti-diabetic effects [53]. These examples prove that although different microbes metabolize substrates via distinct chemical pathways, they can reach the same functional results. In short, the type of microbe determines how the change happens, but the plant’s own chemistry determines the maximum potential benefits.

## 5. Potential Applications of Postbiotic-Oriented Systems Derived from Fermented Medicinal Plant Extracts

Fermented medicinal plant extracts are increasingly positioned as postbiotic-oriented preparations: (i) they can be stabilized as cell-free supernatants, inactivated biomass-containing preparations, or standardized fermented extracts; (ii) their functionality is typically linked to fermentation-derived metabolites and/or inanimate microbial components rather than live microbial persistence; and (iii) they are compatible with industrial requirements for shelf life, safety, and batch-to-batch reproducibility. This application logic aligns with the ISAPP consensus definition of postbiotics as “a preparation of inanimate microorganisms and/or their components that confers a health benefit on the host” [11].

Food and beverage sector: In food systems, fermented medicinal plant extracts are primarily developed as (i) functional beverages/ingredients and (ii) natural preservation or safety-enhancing systems, where fermentation improves organoleptic acceptability, bioactivity retention, and antimicrobial performance.

Fermentation is widely used to transform plant matrices into beverage formats with improved functional profiles, including increased antioxidant potential, enzyme inhibitory activities, and improved flavour acceptance (a critical barrier for many medicinal plants). A clear example of bioactivity targeting is fermentation of honeysuckle (*Lonicera japonica*) with *Lacticaseibacillus rhamnosus* L08: xanthine oxidase (XOD) inhibitory activity increased ~2.08-fold after 96 h, linked to increases in total phenolics/flavonoids and improved flavour characteristics, illustrating how fermentation can produce a consumer-relevant format while simultaneously strengthening a functionally relevant outcome (hyperuricemia-related XOD inhibition) [28].

Further, fermented plant ingredients are increasingly positioned as “clean-label” antimicrobial systems. In a concrete food application, incorporating fermented garlic (via *Lacticaseibacillus casei*) into lamb marinade inhibited *E. coli* by 95%, *S. aureus* by 99%, and *S. typhimurium* by 98%, and reduced lamb microbial load by 0.5 log CFU/g after 3 days of storage while improving water-holding capacity, texture, juiciness, and acceptance, showing a direct technological pathway from fermentation-derived antimicrobials (acids/peptides/other metabolites) to shelf-life extension and quality maintenance [56]. In the study by Basso et al. (2023) [16], the effect of ultrasound alone and in combination with fermentation on the aflatoxin B_1_ reduction in the wheat flour doughs was investigated. The combination of fermentation with ultrasound for 10 min was determined as the most prominent condition for aflatoxin B_1_ reduction [57].

Health, nutraceutical, and pharmaceutical positioning: In health-oriented product development, fermented medicinal plant extracts are increasingly positioned as stabilized bioactive ingredients obtained through controlled microbial processing. Typical formats include liquid extracts, concentrates, spray-dried powders, or encapsulated preparations, enabling their incorporation into orally administered functional foods, dietary supplements, and nutraceutical formulations [58]. For instance, fermented *Centella asiatica* extract prepared with *Lactobacillus* has been investigated in an inflammatory airway disease model (CSE/LPS-induced), supporting the idea that fermented extracts can act as biofunctional candidates beyond “general wellness” positioning [20]. The broader fermented plant extract literature includes examples of human-oriented functional formulations and early clinical claims, underscoring that translation into health contexts has been attempted (with variable rigor depending on study design) [8,58].

Cosmetic and personal care sector: The cosmetic sector represents the most advanced translational domain for postbiotic systems, largely due to the preference for stable, non-viable bioactive ingredients over live microorganisms. Recent reviews emphasize that postbiotics are particularly suitable for topical use because their biological effects persist after microbial inactivation, enabling compatibility with preservatives, conventional formulations, and a long shelf life [59]. A widely cited clinical example is LactoSporin^®^, a preparation composed of extracellular metabolites derived from *Bacillus coagulans*, which was evaluated in a randomized comparative clinical study using a 2% (*w*/*w*) topical cream for 21 days in subjects with mild-to-moderate acne. The study demonstrated significant improvements in acne severity and inflammatory parameters, with efficacy attributed to postbiotic metabolites rather than microbial viability, positioning such preparations as dermatology-adjacent cosmetic active compounds [60]. Beyond acne-oriented formulations, cosmetic postbiotic reviews report in vivo-tested outcomes relevant to wound healing, skin barrier repair, anti-inflammatory activity, and hair-related indications, including alopecia-oriented formulations. These studies demonstrate accelerated wound closure, modulation of inflammatory responses, and improvements in skin hydration and barrier function, indicating that the sector has progressed beyond concept-level claims toward product-format testing and intellectual property development [59]. Within this landscape, fermented medicinal plant extracts are particularly well aligned with cosmetic postbiotic applications through two complementary routes. First, fermentation generates fermented botanical active compounds, in which the biotransformation of phenolics and flavonoids improves extractability, reduces irritation potential, and enhances antioxidant and anti-inflammatory efficacy. Second, fermented plant systems can be formulated as postbiotic-oriented preparations, such as cell-free supernatants or inactivated biomass-containing extracts, combining fermentation-derived microbial metabolites with biotransformed phytochemicals to modulate skin barrier function, inflammation, and microbiome balance without reliance on viable microorganisms [61].

Animal feed, livestock, poultry, and aquaculture: Animal nutrition represents one of the most compelling proof-of-use domains for fermented medicinal plants positioned as postbiotic-oriented feed additives. In this sector, efficacy can be quantitatively assessed through growth performance, feed conversion efficiency, gut morphology, and immune and oxidative stress markers, as well as product quality attributes. Importantly, the reported benefits are consistently associated with the fermented plant preparation itself, reflecting the activity of fermentation-derived metabolites and biotransformed plant matrices [5]. In poultry systems, multiple studies demonstrate that fermented medicinal plants improve performance and health outcomes. For example, supplementation with fermented *Artemisia argyi* in broiler diets has been associated with enhanced growth performance and improved meat quality parameters, including oxidative stability [62]. Similarly, fermented *Astragalus* powder has been shown to improve growth performance, enhance antioxidant status, and stimulate immune function, alongside favourable modulation of intestinal microbiota composition [63]. Additional work in rooster models indicates that fermented *Artemisia argyi* also supports intestinal barrier integrity, anti-inflammatory responses, and microbiota profiles consistent with improved health status [64]. In swine production, fermented multi-herb medicinal plant mixtures have been widely investigated. Dietary inclusion of such fermented Chinese herbal preparations in growing pigs has been associated with improved growth performance, enhanced immune function, and modulation of colon microbiota linked to metabolic and health outcomes [65]. In weaned piglets, supplementation with fermented Chinese herbal medicine feed additives significantly increased average daily gain and feed efficiency while improving intestinal microbiota profiles, supporting better health status and productive performance [57]. These findings demonstrate that fermented herbal feed strategies can enhance both animal health and production efficiency. Aquaculture systems further illustrate the versatility of fermentation-based bioprocessing for feed innovation. Recent studies summarized in the literature report that fermented spent coffee grounds, when used as functional feed ingredients in African catfish, significantly improved growth performance, intestinal morphology, and health-related blood biochemical parameters [66]. This example demonstrates that fermentation can convert low-value plant by-products into cost-effective functional feed materials, expanding the applicability of fermented medicinal and botanical substrates beyond conventional livestock systems.

## 6. Future Perspectives

Current evidence confirms that in vitro fermentation is an effective bioprocessing strategy for modifying medicinal plant extracts and generating stable, postbiotic-oriented bioactivity. However, future progress in this field will depend on addressing several unresolved limitations:

(i) Greater mechanistic clarity is required. Many studies report changes in phytochemical profiles and associated bioactivity, yet the specific enzymatic pathways responsible for these transformations are often insufficiently characterized. Future work should therefore focus on linking microbial enzyme activity to defined structural changes in plant metabolites.
(ii) Standardization remains a major challenge. Variability in plant material, extraction methods, fermentation conditions, and post-fermentation handling limits reproducibility and cross-study comparisons. More consistent reporting of fermentation parameters and chemical composition of fermented extracts is needed to support their development as reliable functional ingredients.
(iii) Translation toward human relevance remains limited. While the biological effects of fermented extracts are well documented in vitro and in animal models, human data are scarce. Importantly, future studies should evaluate fermented extracts as cell-independent preparations, avoiding conceptual overlap with probiotic or gut microbiota-driven effects.
(iv) Clearer regulatory and application-oriented positioning will be required. Fermented medicinal plant extracts currently occupy an intermediate space between traditional herbal preparations and modern functional ingredients. Addressing safety, stability, and long-term use will be essential for their broader acceptance.
(v) Quantitative exposure and biological contextualization require further attention. Although fermentation-associated modifications and functional effects are widely reported, realistic dose ranges, digestion stability, bioaccessibility, and systemic availability of transformed metabolites are rarely assessed. Even when animal models are employed, administered doses are infrequently linked to pharmacokinetic or tissue-distribution analyses. Consequently, functional outcomes demonstrated under controlled experimental conditions are difficult to interpret within physiologically relevant exposure scenarios. Integrating digestion modelling, dose–response evaluation, and pharmacokinetic assessment will be critical for advancing these systems toward biologically contextualized and application-ready formulations.

Overall, future research should move beyond descriptive bioactivity reporting toward predictive, mechanism-informed, quantitatively contextualized, and standardized fermentation models. Such an approach will facilitate the transition of fermented medicinal plant extracts from exploratory experimental systems to well-defined, application-ready, postbiotic-oriented products.

## Figures and Tables

**Table 1:** Inclusion and exclusion criteria applied during screening and full-text evaluation.

Selection Stage	Inclusion Criteria	Exclusion Criteria
**Title and abstract screening**	Studies addressing the fermentation or biotransformation of medicinal plants or herbal extracts	Studies focused solely on gut microbiota, probiotic survival, or clinical microbiome outcomes
	Defined microbial involvement mentioned in the title or abstract	Absence of microbial or fermentation-related context
**Full-text evaluation**	Controlled fermentation or bioprocessing of medicinal plant matrices	Non-fermented extracts or purely chemical modification
	Use of identified microbial strains or consortia	Undefined microbial communities or spontaneous gut fermentation
	Characterization of transformed metabolites (chemical, functional, or biological)	Studies reporting only CFU counts or general bioactivity without transformation analysis
	Sufficient methodological detail enabling comparison	Insufficient experimental description or unclear fermentation conditions

**Table 2:** State of the art on microbial platforms, medicinal plants, dominant biotransformation pathways, and postbiotic-oriented functional outcomes reported in the literature.

Medicinal Plant-Based Substrate	Microbial Platform	Dominant Biotransformation(s)	Main Functional Outcomes	Ref.
*Centella asiatica* extract	*Lactobacillus plantarum*	Phenolic and flavonoid remodelling	Enhanced anti-inflammatory activity	[20]
*Berberis vulgaris* root	*Lactobacillus delbrueckii* and *Lacticaseibacillus rhamnosus*	Alkaloid and phenolic remodelling (berberine-related pathways)	Enhanced antioxidant and antimicrobial activity	[21]
*Leonurus japonicus*	*Saccharomyces* *cerevisiae*	Phenolic remodelling	UVB-induced skin damage protection	[22]
*Atractylodes japonica* rhizome extract	*Lacticaseibacillus* *paracasei*	Phytochemical remodelling	Attenuation of food allergy response (gliadin-induced)	[23]
*Malva sylvestris* extract	LAB	Polyphenol/flavonoid remodelling	Enhanced antioxidant and functional bioactivity	[24]
*Astragali radix* broth	LAB + *Chlorella* Growth Factor	Selective enrichment of polysaccharides and saponins	Enhanced antioxidant and immunomodulatory potential	[25]
Amomum xanthioides	*Lactobacillus casei*	Remodelling of secondary metabolites (phenolic and related constituents)	Amelioration of metabolic dysfunction in high-fat diet-induced obese mice	[26]
Milk thistle and liquorice root extract	*Lactiplantibacillus plantarum*	Flavonolignan and triterpenoid remodelling	Enhanced antioxidant and functional effects	[27]
*Schisandra sphenanthera* fruit	*Lactiplantibacillus plantarum*	Polysaccharide release and structural remodelling	Enhanced functional polysaccharide properties	[26]
*Lonicera japonica Thunb*. (leaves)	*Lacticaseibacillus* *rhamnosus*	Phenolic acid remodelling; flavonoid transformation; volatile profile remodelling	Enhanced xanthine oxidase inhibitory activity; improved sensory profile	[28]
*Ginkgo biloba* leaves	SSF *Bacillus subtilis* + pulsed light	Detoxification of ginkgolic acids; matrix restructuring	Improved safety and bioactive profile	[29]
Ginger rhizome	*Bifidobacterium adolescentis* + *Monascus purpureus*	Targeted gingerol biotransformation; polyphenol remodelling	Enhanced antioxidant activity	[30]
Saffron petals (*Crocus sativus* L.)	*Saccharomyces cerevisiae*	Matrix restructuring and anthocyanin release	Increased anthocyanin availability	[31]
*Paeonia lactiflora* root	*Saccharomycopsis* *fibuligera*	Broad phytochemical enrichment	Enhanced antioxidant capacity	[32]
*Dendrobium officinale* leaves	*Aspergillus oryzae*	Deglycosylation of flavonoids; aglycone enrichment	Enhanced antioxidant activity	[33]
Okra juice	*Lactiplantibacillus plantarum*	Polysaccharide depolymerization and structural modification	Improved immunomodulatory activity	[34]
*Diaphragma juglandis* fructus	*Aspergillus niger*	Matrix-level polyphenol release	Increased biological activity	[35]
*Elaeocarpus sylvestris* hot-water extract	*Pichia fermentans* and *Saccharomyces cerevisiae*	Reprogramming of organic acid metabolism	Altered metabolic and volatile profile	[36]
*Toona sinensis* buds	*Lactiplantibacillus plantarum*	Flavonoid and alkaloid remodelling	Enhanced metabolic enzyme inhibition	[37]
*Orthosiphon stamineus* leaves	*Saccharomyces cerevisiae*	Phenolic release and extractability enhancement	Increased antioxidant activity	[38]
*Inula britannica*	*Lactobacillus plantarum*	Selective catechin (EGCG) enrichment; detoxification	Reduced toxicity; improved bioactivity	[39]
Rose–shiitake blended beverage	Multiple LAB strains	Broad matrix remodelling (phenolics, amino acids)	Enhanced antioxidant activity	[40]
Mulberry leaves	*Monascus purpureus*	Flavonoid diversification; glycoside conversion	Improved bioaccessibility	[41]
Pigeon pea roots	*Penicillium rubens*	Isoflavone biotransformation (genistein release)	Increased phytoestrogenic potential	[42]
*Phyllostachys glauca* leaf juice	*Streptococcus thermophilus*	Polysaccharide restructuring	Polysaccharide restructuring	[43]
Mulberry leaf powder	*Pediococcus pentosaceus*	GABA biosynthesis; phytic acid degradation	Improved antioxidant profile; reduced antinutrients	[44]
Mulberry leaves	Co-fermentation with *Aspergillus cristatus*	Flavonoid remodelling	Increased α-glucosidase inhibition	[45]
Wolfberry–longan juice	Co-fermentation with *Lacticaseibacillus paracasei* and *Lactococcus lactis subsp*. *lactis*	Phenolic enrichment in fermented plant-based matrix	Enhanced antioxidant capacity	[46]
*Ulmus davidiana* root bark	*Bacillus licheniformis*	Phenolic restructuring; antimicrobial compound release	Enhanced antioxidant and antimicrobial activity	[47]
*Perilla frutescens* leaves	LAB	Metabolome-level phytochemical remodelling	Enhanced antioxidant/metabolic bioactivity	[48]
*Cinnamomum cassia*	*Lactiplantibacillus plantarum*	Phenolic remodelling (limited mechanistic resolution)	Increased antioxidant activity	[49]
Wild turmeric (*Curcuma aromatica*)	*Rhizopus oligosporus*	Curcuminoid biotransformation and release	Enhanced antioxidant activity	[50]
Jujube juice	*Lactobacillus acidophilus*, *Lactobacillus casei*, *Lactobacillus helveticus*. and *Lactobacillus plantarum*	Quantitative phenolic enrichment	Improved antioxidant capacity	[51]
Moringa leaves flour	*Aspergillus niger*	Deep fungal biotransformation; novel metabolite formation	Enhanced antioxidant and antimicrobial activity	[52]
Mulberry leaves	*Monascus anka*	Flavonoid deglycosylation	α-Glucosidase inhibition	[53]
Seven traditional Chinese herbal extracts	*Lactobacillus rhamnosus*	Phytochemical remodelling affecting melanogenesis pathways	Anti-pigmentation (CREB/MITF/tyrosinase regulation)	[54]
*Andrographis paniculate* extract	*Aspergillus oryzae*	Biotransformation of diterpenoid lactones (oxidation, hydroxylation, structural modification)	Generation of novel andrographolide derivatives with diversified bioactive potential	[55]

## Data Availability

The original contributions presented in the study are included in the article; further inquiries can be directed to the corresponding author.
